# T Cell Hypo-Responsiveness against *Leishmania major* in MAP Kinase Phosphatase (MKP) 2 Deficient C57BL/6 Mice Does Not Alter the Healer Disease Phenotype

**DOI:** 10.1371/journal.pntd.0002064

**Published:** 2013-02-21

**Authors:** Juliane Schroeder, H. Adrienne McGachy, Stuart Woods, Robin Plevin, James Alexander

**Affiliations:** Strathclyde Institute for Pharmacy & Biomedical Sciences, University of Strathclyde, Glasgow, United Kingdom; Institut Pasteur, France

## Abstract

We have recently demonstrated that MAP kinase phosphatase 2 (MKP-2) deficient C57BL/6 mice, unlike their wild-type counterparts, are unable to control infection with the protozoan parasite *Leishmania mexicana*. Increased susceptibility was associated with elevated Arginase-1 levels and reduced iNOS activity in macrophages as well as a diminished T_H_1 response. By contrast, in the present study footpad infection of MKP-2^−/−^ mice with *L. major* resulted in a healing response as measured by lesion size and parasite numbers similar to infected MKP-2^+/+^ mice. Analysis of immune responses following infection demonstrated a reduced T_H_1 response in MKP-2^−/−^ mice with lower parasite specific serum IgG2b levels, a lower frequency of IFN-γ and TNF-α producing CD4^+^ and CD8^+^ T cells and lower antigen stimulated spleen cell IFN-γ production than their wild-type counterparts. However, infected MKP-2^−/−^ mice also had similarly reduced levels of antigen induced spleen and lymph node cell IL-4 production compared with MKP-2^+/+^ mice as well as reduced levels of parasite-specific IgG1 in the serum, indicating a general T cell hypo-responsiveness. Consequently the overall T_H_1/T_H_2 balance was unaltered in MKP-2^−/−^ compared with wild-type mice. Although non-stimulated MKP-2^−/−^ macrophages were more permissive to *L. major* growth than macrophages from MKP-2^+/+^ mice, reflecting their reduced iNOS and increased Arginase-1 expression, LPS/IFN-γ activation was equally effective at controlling parasite growth in MKP-2^−/−^ and MKP-2^+/+^ macrophages. Consequently, in the absence of any switch in the T_H_1/T_H_2 balance in MKP-2^−/−^ mice, no significant change in disease phenotype was observed.

## Author Summary

*Leishmania* species are parasites that are of extensive public health importance in the tropics and subtropics. Within humans the parasites are intracellular and reside particularly within macrophages. Classical activation of macrophages by Interferon-γ (IFN-γ) induces the enzyme nitric oxide synthase (iNOS) and parasites are killed via the production of nitric oxide (NO) from the substrate L-arginine. Alternative activation by Interleukin-4 (IL-4) results in Arginase-1 expression, which depletes L-arginine and facilitates parasite growth. We have recently shown that MAP Kinase Phosphatase-2 (MKP-2) suppresses macrophage Arginase-1 and that C57BL/6 mice with a deletion of this gene are subsequently extremely susceptible to New World cutaneous leishmaniasis caused by *Leishmania mexicana*. Surprisingly, MKP-2 deficient mice have been shown here to be resistant to Old World cutaneous leishmaniasis caused by *L. major*. We show that during infection with *L. major*, enhanced Arginase-1 in MKP-2 deficient mice serves to induce a generalized T cell hypo-responsiveness so that IFN-γ and IL-4 levels are equally suppressed compared with intact mice. In addition, unlike *L. mexicana*, classically activated MKP-2 deficient macrophages were able to control *L. major* growth equally as well as MKP-2 intact macrophages, highlighting a fundamental difference in the control of these two species.

## Introduction

*Leishmania* species are protozoan parasites that are transmitted by infected female sandflies and cause a wide spectrum of diseases ranging from self-healing cutaneous lesions to fatal systemic disease. After entering their vertebrate host, promastigotes are taken up initially by neutrophils and ultimately macrophages and dendritic cells, where they turn rapidly into amastigotes and survive within parasitophorous vacuoles [1]. Resistance against cutaneous infection with *Leishmania (L.) major* typically requires the presence of an antigen-specific type 1 immune response comprising of IFN-γ/TNF-α/IL-2 producing CD4^+^ T [2], [3] cells but also CD8^+^ T cells have been shown to play an important role in parasite clearance [4], [5]. Subsequently, activated T cells migrate to the site of infection where they release IFN-γ and TNF-α which in turn upregulate inducible nitric oxide synthase (iNOS) in infected macrophages, enabling nitric oxide (NO) mediated killing of the intracellular parasites [6], [7]. Susceptibility, on the other hand, has been associated with a failure to produce a type-1 response, which may be a consequence of IL-10 production from Fc-γ mediated macrophage uptake of amastigotes [8], or from natural and or type-1 regulatory T cells [9]–[11], or regulatory B cells [12], or an elevated T_H_2 response and the excessive production of IL-4 by CD4^+^ T cells [2], [13], or indeed a combination of all these factors (reviewed by [14]). IL-4 in particular has been shown to promote alternative macrophage activation including increased expression of Arginase-1 [15], suppression of iNOS [16] and increased growth of *L. major*.

Mitogen-activated protein kinase phosphatase 2 (MKP-2) is a dual-specific nuclear phosphatase (DUSP) and is associated with the MAPK signalling pathway, where it has been shown to dephosphorylate and thereby inactivate protein kinases ERK, JNK but not p38 in vitro [17], [18]. MKP-2 could therefore have significant effects on *L. major* infection as these parasites have a well recognized ability to subvert the development of T_H_1 responses partly via effects upon MAP kinase signalling. Studies using *L. major* metacyclic promastigotes indicated that the parasite via lipophosphoglycan (LPG) differentially regulated IL-12 as well as NO production by targeting ERK and p38 MAPK, respectively [19], [20]. In order to better understand the role of MKP-2 in immune functions we, and others, have recently created MKP-2 deficient mice on a C57BL/6 background [21]–[24]. Thus regulatory roles specific for MKP-2 have been demonstrated in the inflammatory response associated with sepsis [22], cell cycle progression and apoptosis [23] and infection [21]. Furthermore, MKP-2^−/−^ macrophages have severe ablation of LPS or IFN-γ -induced iNOS expression and nitric oxide release and enhanced basal expression of Arginase-1. Given that Arginase-1 competes with iNOS for their common substrate L-arginine, it suggested that MKP-2 could have a regulatory function significant in immune responses involving intracellular pathogens [25]. Indeed, following infection with the intracellular parasite *L. mexicana* we demonstrated that it was changes in Arginase-1 and iNOS rather than changes in kinase mediated signalling that dictated the subsequent in vivo response to MKP-2 deletion [21]. MKP-2^−/−^ mice displayed increased lesion size and parasite burden, and a significantly modified T_H_1/T_H_2 bias compared with wild-type counterparts [21]. This was related to a significant down-regulation of specific T_H_1 activity in MKP-2 deficient animals. However, there was no intrinsic defect in MKP-2^−/−^ T cell function as measured by anti-CD3 induced IFN-γ production. Rather, MKP-2^−/−^ bone marrow-derived macrophages, as a consequence of increased Arginase-1 expression, were found to be inherently more susceptible to infection with *L. mexicana* than MKP-2^+/+^ derived macrophages.

The immune-regulatory mechanisms controlling *L. mexicana* and *L. major* differ significantly and while most mouse strains heal following infection with *L. major* the vast majority develop chronic infections following infection with *L. mexicana* (reviewed by [26]). Thus, while C57BL/6 mice can control *L*. *mexicana* lesion growth they do not cure and MKP-2 deficiency results in progressive disease following *L. mexicana* infection [21]. C57BL/6 mice on the other hand are more resistant to *L. major* and lesions heal following infection. Given the well documented importance of NO killing in controlling *L. major* infections we addressed the question as to whether MKP-2 deficiency would make C57BL/6 mice more susceptible to this parasite. To our surprise no distinct disease phenotype was noted in MKP-2^−/−^ mice infected with this parasite. Although type-1 responses in infected MKP-2^−/−^ mice were down-regulated, type-2 responses were equally suppressed and the T_H_1/T_H_2 balance remained unaffected. Furthermore although MKP-2^−/−^ naïve macrophages were more permissive host cells for *L. major* than wild-type macrophages, if classically activated they were equally effective at controlling parasite growth.

## Materials and Methods

### Ethics statement

Female DUSP-4 (MKP-2) wild-type and deficient mice were generated as previously described and bred onto C57BL/6 background [21] and female BALB/c mice were bred and maintained under specific pathogen-free conditions in the animal facilities of the Strathclyde Institute for Pharmacy and Biomedical Sciences at the University of Strathclyde. Animals were used at 6–9 weeks of age and were age matched within each experiment. All animal experiments adhered to the UK Animals (Scientific Procedures) Act 1986 and were conducted under Project Licenses to RP (PPL60/3439 “genetic models of cancer and inflammation”) and JA (PPL60/3929 “mechanism of control of parasite infection”) granted by the UK Home Office and with local ethical approval.

### Parasite infections

*Leishmania major* (IR75) promastigotes were grown in HOMEM (Gibco) supplemented with 10% FCS (Biosera, East Sussex, UK) until late stationary phase. In order to enable direct comparison with previous studies on the role of MKP-2 during *L. mexicana* infections [21] and to Arginase-1/*L. major* studies [27], [28] relevant to the work presented here we used the high dose inoculation model. Mice were given 2×10^6^ *L. major* subcutaneously into the left hind foot pad and lesion development was monitored as the difference in thickness between infected and uninfected foot pads using a dial gauge calliper. For determination of parasite burden, mice were sacrificed by cerebral dislocation and foot pads, popliteal lymph nodes and spleens were removed and homogenized. Single cell suspensions were adjusted to equal volumes and subjected to limiting dilution assay as described elsewhere [29].

### T cell assay

Single cell suspensions from spleens and lymph nodes of infected mice were prepared and erythrocytes were lysed using Erythrocyte lysis buffer pH 7.3 (160 mM NH_4_Cl, 10 mM KHCO_3_ and 100 µM EDTA). Cells were resuspended in RPMI containing 10% FCS, L-Glutamine and Penicillin/Streptomycin and 2×10^6^ splenocytes were restimulated either on dendritic cells pulsed with 5 µg *L. major* antigen over night, PMA (10 ng/ml)/Ionomycin (500 ng/ml), ConA (10 µg/ml) or medium alone for 6 h (intracellular staining) or 48–72 h (ELISA) in 24-well plates. For intracellular staining, cytokine release was inhibited by the addition of BrefeldinA (10 µg/ml) at 3 h into re-stimulation. All reagents were supplied by Sigma-Aldrich (St. Louis, USA).

### Magnet assisted cell sorting (MACS)

CD4^+^ T cells from spleens and lymph nodes of infected mice were isolated on LS magnetic columns (Miltenyi Biotec GmbH, Bergisch Gladbach, Germany) using the negative selection kit (Miltenyi) following manufacturer's instructions.

### Enzyme linked immunosorbant assay (ELISA)

Mouse serum was analysed individually for antigen-specific IgG1 or IgG2b using single sided ELISA as previously described [30]. Resulting titres were expressed as reciprocal values of the half-maximal absorption at 450 nm using a Spectromax 190 plate reader.

Cytokines from T cell supernatants were determined with Sandwich-ELISA using cytokine specific capture and detection antibodies (BD Bioscience) and recombinant cytokines for standard curves (R&D Systems, Minneapolis, USA). IL-4 was determined using the mouse IL-4 Quantikine kit (R&D Systems) following manufacturer's instructions. To allow detection of IL-4, 5 µg/ml IL-4 receptor blocking antibody (anti-mouse CD124, BD Pharmingen, USA) was added to the cultures.

### Flow cytometry

Cells were harvested and passed through a nitex mesh to remove clumps. For isotype control, 5×10^5^ cells of medium-, antigen- and PMA/Ionomycin-restimulated splenocytes were pooled for each individual. After blocking unspecific binding with 10% mouse serum and Fc receptor blocking antibodies (anti-mouse CD16/32, eBioscience, UK), cell surface was stained with conjugated antibodies for CD3e (PerCP), CD4 (APC-H7), both BD Pharmingen (USA) and CD8b (Alexa Fluor 488, eBioscience UK) for 45 min at 4°C. Cells were fixed for 15 min and permeabilized using the Fix and Perm kit (Invitrogen, Paisley, UK). Intracellular staining was carried out simultaneously with permeabilisation for 1 h at room temperature using conjugated antibodies for IFN-γ (Allophycocyanin (APC)) and TNF-α (Phycoerythrin (PE)) or their respective isotypes anti-rat IgG1 (APC) and anti-rat IgG1 (PE), all eBioscience. After washing steps, cells were resuspended in 200 µl PBS and subsequently run and analysed on the FACS Canto flow cytometer (BD Bioscience) using FACS Diva software.

### Cell culture

Bone marrow-derived macrophages (BMDM) and dendritic cells were derived from tibia and femur of 6 to 8 week old mice. Bone marrow was flushed, and in order to obtain macrophages, cells were resuspended in DMEM containing 10% foetal calf serum (FCS), 30% L929-conditioned medium, 2 mM L-Glutamine (Gibco, Invitrogen, Paisley, UK), 1% Penicillin/Streptomycin (Gibco) and seeded into Petri dishes. After 10 days at 37°C, adherent macrophages were harvested with cold/warm PBS, washed and resuspended in complete RPMI (10% FCS, 2 mM L-Glutamine, 1% Penicillin/Streptomycin). Dendritic cells were generated from bone marrow precursors by culturing in RPMI containing 10% FCS, 2 mM L-Glutamine, 1% Penicillin/Streptomycin and 2.5–10% GM-CSF conditioned medium (X63). Non-adherent cells were harvested at day 7, washed and resuspended in complete RPMI.

### Macrophage infections

BMDMs were seeded onto cover slips at 2×10^5^ cells/500 µl in 24-well plates and left to adhere at 37°C over night. For infection *L. major* promastigotes (IR75) were added at a multiplicity of infection (MOI) of 5 and plates were briefly spun at 300×g to allow close proximity of parasite and macrophages. After 1 h at 34°C, supernatants were removed and macrophages were washed in PBS to remove any free parasites. Fresh complete RPMI supplemented with or without 100 ng/ml LPS, 100 U/ml IFN-γ or 100 U/ml IL-4 was added and cells were incubated at 34°C for different periods of time.

To determine number of parasites and infection rates, macrophages were fixed in methanol and stained with Giemsa solution. Cover slips were mounted onto glass slides and intracellular parasites were counted in a total of 200 macrophages across each cover slip using a bright field microscope.

### Immunoblotting

Arginase-1 expression was determined from whole cell lysates of 1×10^6^ *L. major*-infected BMDM. SDS-PAGE analysis and detection was carried out as described elsewhere [21].

### Statistics

Statistical analysis was performed using GraphPad Prism Program (Version 4.0, GraphPad Software, San Diego, California). P values below or equal to 0.05 were considered significant.

## Results

### MKP-2 deficiency in C57BL/6 mice did not alter the course of infection with *L. major*

**Figure 1:**
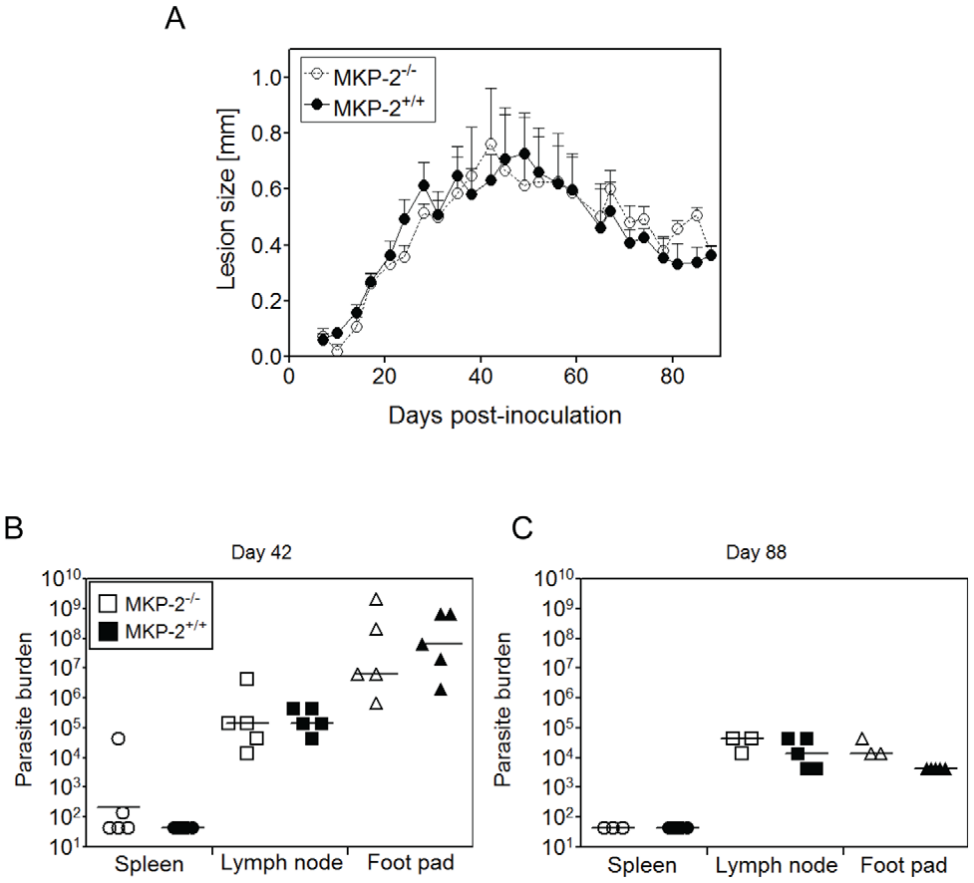
MKP-2 deficiency does not alter the course of infection with *L. major*. Ten MKP-2^−/−^ (open symbols) and ten MKP-2^+/+^ mice (closed symbols) were given with 2×10^6^ *L. major* promastigotes subcutaneously into the left hind foot pad. The right uninfected foot pad was used for reference. Lesion development was monitored by measuring the footpad thickness (A) over time using a dial gauge calliper. At peak of infection (day 42, B) and after the onset of healing (day 88, C) five mice were sacrificed at random and parasite burdens in foot pad, popliteal lymph nodes and spleens were determined using a limiting dilution assay. Detection limit was 65 for spleens and 6500 for lymph node and foot pads. Error bars are shown as standard error of the mean (SEM). The data shown is representative of at least three independent experiments.

MKP-2 deficient and MKP-2 wild-type C57BL/6 mice were given 2×10^6^ late-stationary phase *L. major* promastigotes subcutaneously into the left hind footpad and lesion development was monitored over 12–13 weeks. Surprisingly, given that MKP-2^−/−^ mice were previously found to be more susceptible to *L. mexicana* than their wild-type counterparts [21], no difference in lesion growth between MKP-2^−/−^ and MKP-2^+/+^ mice was detected throughout the course of infection with *L. major*. Both MKP-2^−/−^ and MKP-2^+/+^ mice developed lesions which healed spontaneously after several weeks and in a manner typical for *L. major* resistant C57BL/6 mice (Figure 1A). At two time points, the peak of infection (Figure 1B) and after onset of healing (Figure 1C), five mice were sacrificed and the parasite burdens of infected footpads, popliteal draining lymph nodes and spleens were determined. Consistent with the lesion development, parasite numbers were high at the peak of infection and consequently dropped with the onset of healing. We could not observe a difference in phenotype between wild-type and MKP-2^−/−^ mice at either peak lesion growth or after healing in any of the tissues examined. This observation was made in several independent experiments.

### Enhanced susceptibility of MKP-2^−/−^ macrophages to infection with *L. major* is ablated following classical but not innate or alternative activation

**Figure 2:**
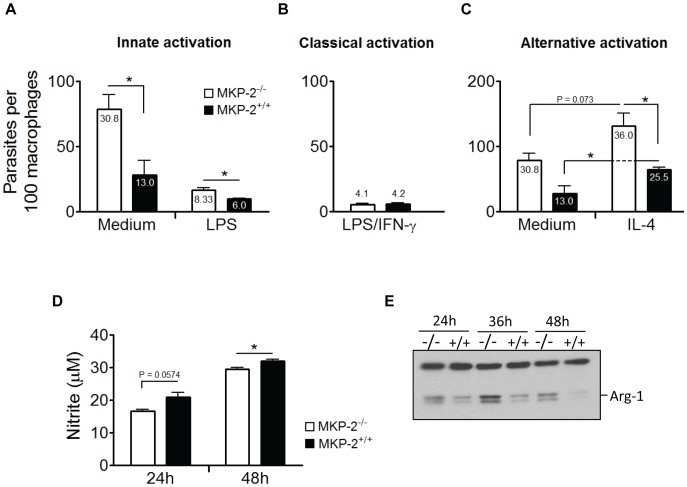
Macrophages of MKP-2^−/−^ mice are inherently more susceptible to *L. major* parasites. Bone marrow-derived macrophages of MKP-2^−/−^ (open symbols) and MKP-2^+/+^ (closed symbols) grown on cover slips were infected with *L. major* promastigotes at a multiplicity of infection (MOI) of 5. One hour after infection free parasites were removed by washing and medium was replaced with complete RPMI supplemented with or without 100 ng/ml LPS or a combination of 100 ng/ml LPS and 100 U/ml IFN-γ, or 100 U/ml IL-4 and left for 24 or 48 h at 34°C (A, B and C respectively). Macrophages were fixed in methanol and stained with Giemsa. Intracellular parasites were counted in a total of 200 macrophages using a bright field microscope and are shown as number of parasites per 100 macrophages. The mean percentage of infected macrophages is also expressed as numbers inside the columns. At time points indicated supernatants from LPS-stimulated macrophages were removed and tested for nitric oxide production using Griess reagent (D). Error bars show standard error of the mean (SEM). The data shown is representative of at least three independent experiments. For analysis of Arginase-1 expression 1×10^6^ BMDM were infected at an MOI of 5 and whole cell lysates were run on 12% gels, blotted on nitrocellulose membranes and stained for Arginase-1. The top cross-reactive band serves as loading control (E).

Our previous studies demonstrated that the susceptibility of MKP-2 deficient mice is to a significant degree due to MKP-2^−/−^ macrophages being inherently more susceptible to the growth of *L. mexicana* than wild-type macrophages a dichotomy that was maintained even following classical activation [21]. We therefore examined the infectivity of *L. major* metacyclic promastigotes for MKP-2^−/−^ and MKP-2^+/+^ naïve, innately, classically or alternatively activated bone marrow derived macrophages (Figure 2 A, B and C). Over several experiments we found that infected non-stimulated MKP-2^−/−^ BMDM cultures maintained significantly increased parasite burdens and increased frequency of infected macrophages than cultures using their wild-type counterparts (Figure 2A). Our previous studies with *L. mexicana* had demonstrated the increased susceptibility of non-stimulated MKP-2^−/−^ macrophages to infection to be related to elevated Arginase-1 levels compared with MKP-2^+/+^ macrophages and similarly enhanced levels of Arginase-1 were found in MKP-2^−/−^ macrophages following infection with *L. major* promastigotes (Figure 2E). Innate macrophage activation with 100 ng/ml LPS greatly decreased the number of intracellular parasites as well as the frequency of infected cells in both MKP-2^−/−^ and MKP-2^+/+^ macrophages (Figure 2A). However, despite activation with LPS, MKP-2 deficient macrophages still presented higher parasite survival than their wild-type counterparts. As nitric oxide (NO) is essential for killing of intracellular parasites, we determined the production of NO by measuring nitrite levels in the supernatants of infected macrophages 24 and 48 h after activation with LPS. Consistent with our previous observations with *L. mexicana*, MKP-2^−/−^ macrophages showed a reduced NO production at both time points in response to LPS activation (Figure 2D). However, whereas MKP-2^−/−^ macrophages classically activated with LPS and IFN-γ failed to reduce *L. mexicana* parasite burdens to the level of similarly treated MKP-2^+/+^ macrophages [21] such treatment sufficed following infection with *L. major* promastigotes to ablate any differences in susceptibility between MKP-2^−/−^ and MKP-2^+/+^ host cells (Figure 2B) and corresponded to LPS and IFN-γ treatment ablating the deficiency in NO production in MKP-2^−/−^ macrophages infected with *L. major* (data not shown). The addition of recombinant IL-4 to macrophages infected with *L. major* doubled the numbers of intracellular parasites in both wild-type and MKP-2^−/−^ macrophages (Figure 2C), highlighting the disease-promoting attributes of IL-4 and alternative macrophage activation in *L. major* infections characterized by elevated Arginase-1 and reduced iNOS expression [16], [31].

### MKP-2 deficiency results in a diminished T_H_1 response following infection with *L. major*

**Figure 3:**
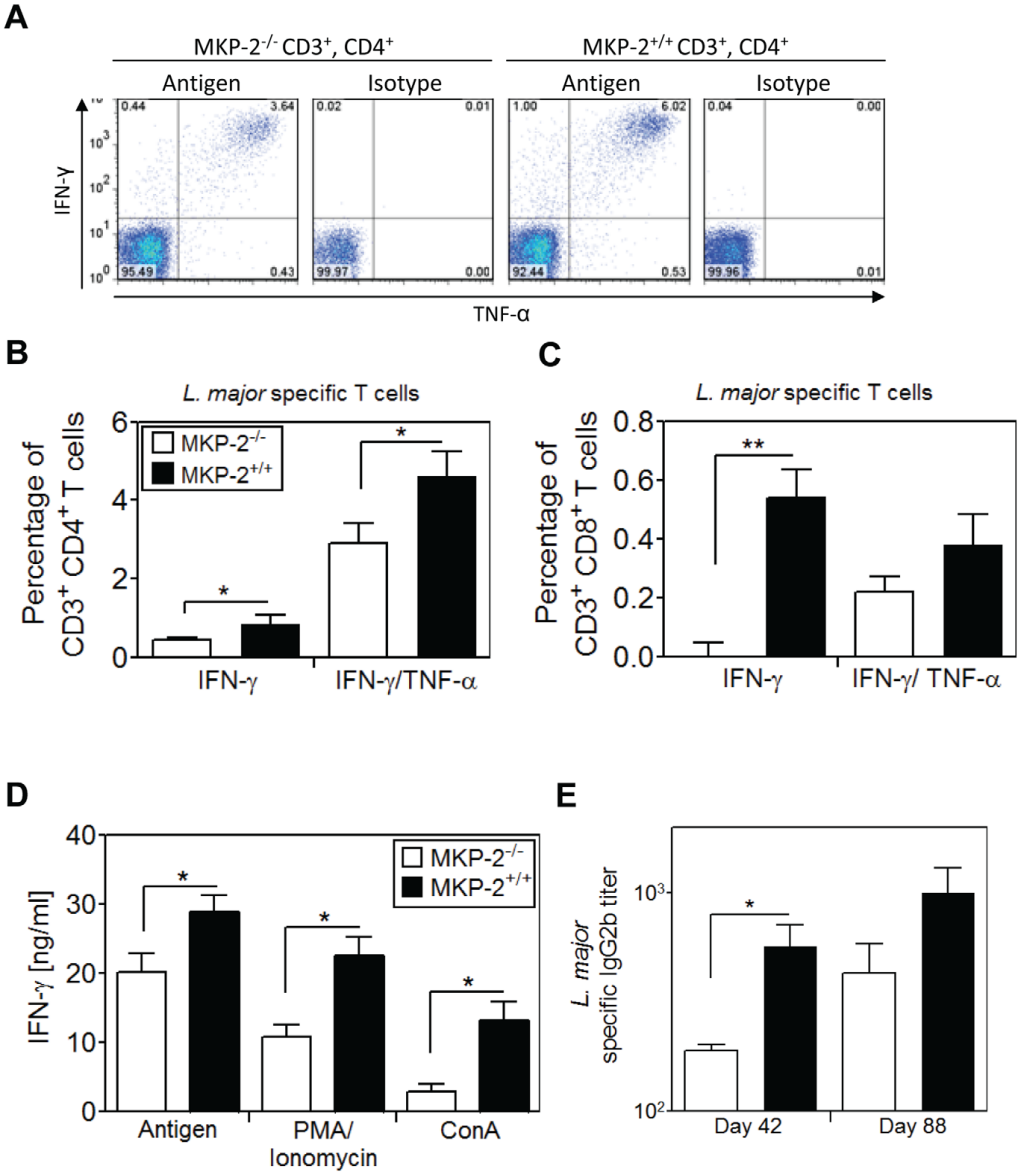
MKP-2 deficient mice infected with *L. major* have a reduced T_H_1 response. Splenocytes from *L. major* infected MKP-2^−/−^ (open columns) and MKP-2^+/+^ (closed columns) mice were re-stimulated either with antigen pulsed DC, PMA/Ionomycin, ConA or with unpulsed DC (Medium control) for 6 h (FACS) or 48 h (ELISA). Cells were stained for T cell surface markers CD3, CD4 and CD8 as well as intracellular cytokines IFN-γ and TNF-α or their respective isotypes (A). Percentages of IFN-γ single and IFN-γ/TNF-α double producing parasite-specific CD4^+^ (B) and CD8^+^ (C) T cells are shown after normalization to medium and isotype controls. Supernatants of 48 h T cell re-stimulations were analyzed for IFN-γ in a sandwich ELISA and values were normalized to medium controls (D). Serum of infected mice was collected and analysed for parasite-specific antibody isotype IgG2b. The titres were calculated as reciprocal dilution of the half-maximal absorption at 450 nm (E). Error bars are shown as standard error of the mean (SEM). *P<0.05, **P<0.01; one-tailed Mann Whitney U test. The data shown is representative of two independent experiments.

As no discernible change in the healing disease phenotype was observed between MKP-2^−/−^ and MKP-2^+/+^ mice infected with *L. major*, and given that classically activated macrophages from MKP-2^−/−^ mice were equally as effective as their wild-type counterparts at killing parasites we compared the type-1 response generated in MKP-2^+/+^ and MKP-2^−/−^ following infection. After re-stimulation with soluble *L. major* antigen-pulsed DCs, flow cytometric analysis of CD4^+^ T cells from the spleens of infected animals demonstrated that MKP-2^−/−^ mice had a significantly lower frequency of IFN-γ single producing and IFN-γ/TNF-α double producing CD4^+^ and CD8^+^ T cells (P<0.05) than their wild-type counterparts (Figure 3A, B and C). Similarly significantly reduced IFN-γ production (P<0.05) was measured in the supernatants of MKP-2^−/−^ derived T cell cultures after stimulation with *L. major* antigen-pulsed DC, PMA/Ionomycin or ConA (Figure 3D). Finally we analysed the serum of *L. major* infected mice for the presence of antigen-specific IgG2b, a surrogate marker for T_H_1 responses, as IFN-γ is essential for IgG class switch to 2a and 2b [32], [33]. At both stages of infection, MKP-2 deficient mice showed lower IgG2b titres, being statistical significant (P<0.05) at day 42 post-infection (Figure 3E). Overall, our data suggest that MKP-2 deficiency results in a diminished T_H_1 response during infection with *L. major*, similar to our previous observations using *L. mexicana* infections [21].

### MKP-2 deficiency results in a reduced T_H_2 response in *L. major* infected mice

**Figure 4:**
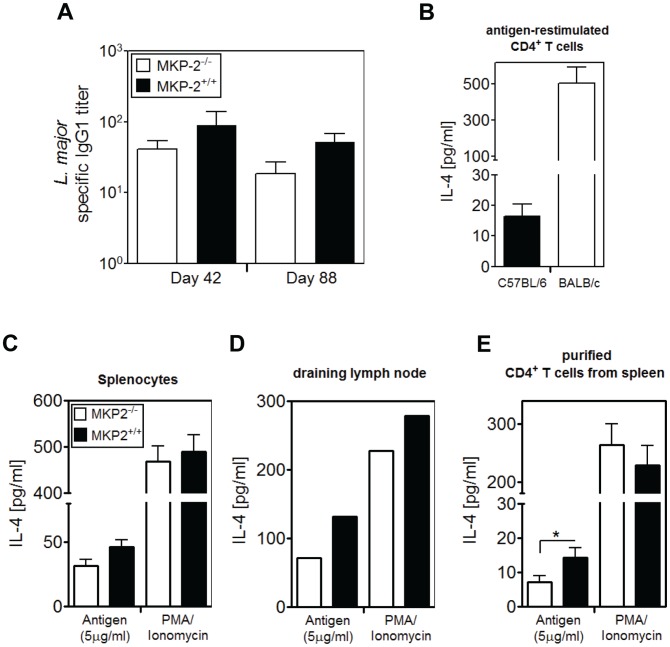
MKP-2 deficient mice infected with *L. major* have a reduced T_H_2 response. Serum of *L. major* infected MKP-2^−/−^ (open column) and MKP-2^+/+^ (closed column) mice was analysed for parasite-specific antibody isotype IgG1 (A). The titres were calculated as reciprocal dilution of the half-maximal absorption at 450 nm. In order to measure T_H_2 responses, 3 MKP-2^−/−^, 3 MKP-2^+/+^ and 3 BALB/c mice were given 2×10^6^ *L. major* promastigotes subcutanteously into the hind foot pad. At peak of infection, mice were sacrificed and spleen and popliteal lymph node removed. Spleens were analysed individually and lymph nodes pooled within each group. CD4^+^ T cells were magnet-purified from spleens using a negative isolation kit. Splenocytes (B, C), lymph node cells (D) and purified CD4^+^ T cells (E) were re-stimulated either with antigen pulsed DC, PMA/Ionomycin, or with unpulsed DC (Medium control) in the presence of IL-4 receptor blocking antibody for 72 h and supernatants were analysed immediately for IL-4. Error bars show standard error of the mean (SEM). *P<0.05 one-tailed unpaired t test.

In stark contrast to experimental infection with *L. mexicana*, MKP-2 deficient mice did not show an increased susceptibility to infections with *L. major* compared with wild-type mice. This was despite a clearly diminished T_H_1 response in MKP-2^−/−^ compared with MKP-2^+/+^ mice following *L. major* infection (Figure 3). However, MKP-2 is a generalized negative regulator of Arginase-1in tissues rich in phagocytes (Figure S1) and it is well documented that Arginase-1 can induce hypo-responsiveness in a pan-T cell fashion [27], [28] [34]. We therefore examined whether the lack of a parasite-specific T_H_1 response could be counterbalanced by concomitant impairment of a T_H_2 response, leaving the relative T_H_1/T_H_2 balance undisturbed. Over a number of experiments we found MKP-2^−/−^ mice infected with *L. major* produced less IgG1 but not significantly less than their similarly infected wild-type counterparts (Figure 4A). To directly measure the parasite induced production of IL-4, we infected MKP-2 wild-type and deficient mice as well as susceptible BALB/c mice with *L. major* promastigotes. At the peak of infection, mice were sacrificed, spleens and draining lymph nodes removed and analyzed for the production of IL-4. Whole spleen and lymph node cells were re-stimulated with soluble *L. major* antigen-pulsed DC, PMA/Ionomycin or ConA for 72 h in the presence of IL-4 receptor blocking antibody to prevent immediate uptake of freshly produced IL-4 in an auto- or paracrine fashion. As expected, susceptible BALB/c mice produced high levels of IL-4 compared with mice from the C57BL/6 background (Figure 4B). However, while antigen induced IL-4 production was reduced in MKP-2^−/−^ splenocytes and draining lymph node cells (Figure 4C and D) compared with wild-type equivalent cell populations this was not clearly significant because of small sample sizes. For a more precise examination of the T_H_2 response we therefore isolated CD4^+^ T cells from spleen suspensions by negative selection and re-stimulated these as described above. The levels of IL-4 produced by CD4^+^ T cells in response to *L. major* infection were indeed clearly and significantly reduced in MKP-2 deficient mice when compared with wild-type mice (Figure 4E), thus confirming a reduced T_H_2 response in infected MKP-2^−/−^ mice.

### The T_H_1/T_H_2 balance in MKP-2 deficient mice compared with wild type mice is sustained by general T cell hypo-responsiveness against *L. major*

**Figure 5:**
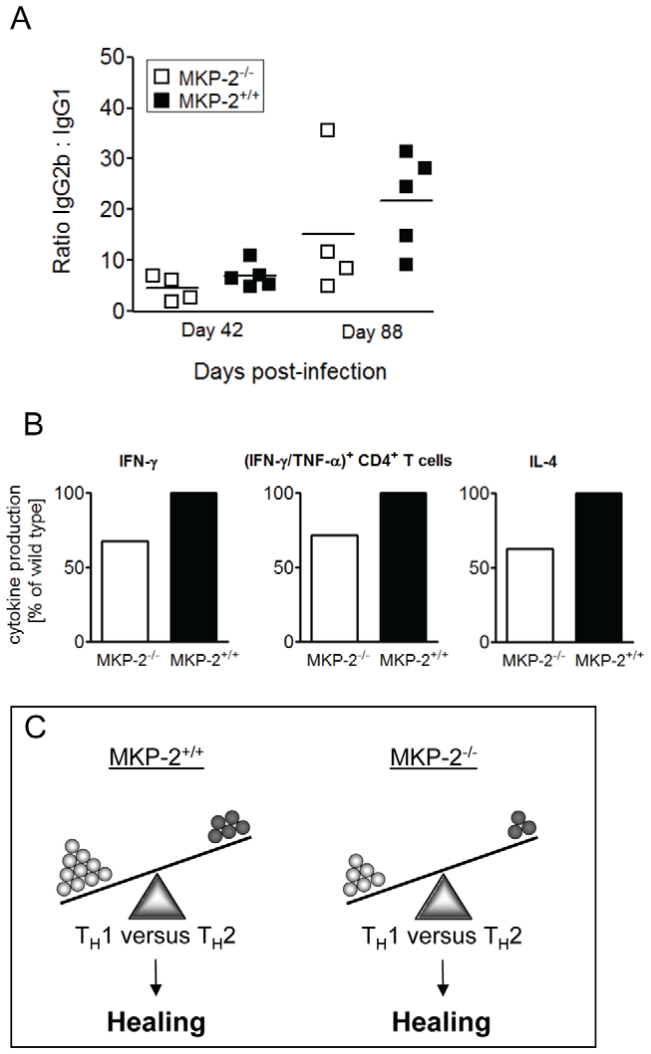
A sustained T_H_1/T_H_2 balance in MKP-2 deficient mice is due to general T cell hypo-responsiveness against *L. major*. Antibody titres for IgG2b were divided by respective titres for IgG1 for each individual mouse and for both time points. Higher values are indicative for higher IgG2b levels and lower values reflect a higher IgG1 response. The bar indicates the median of each group (A). The percent reduction of the mean for each measured parameter (IFN-γ, IL-4 and IFN-γ/TNF-α^+^ CD4^+^ T cells) of MKP-2^−/−^ mice was calculated against the mean values of MKP-2^+/+^ mice ( = 100%) (B). If both immune responses (T_H_1, (IFN-γ/TNF-α^+^ CD4^+^ T cells and IFN-γ) versus T_H_2 (IL-4) are reduced to a similar extend (B), the balance between disease promoting T_H_2 and the protective T_H_1 response is not disturbed. Since the mice are on a C57BL/6 background the T_H_1 response will outweigh the T_H_2 response and consequently result in a healing phenotype (C).

Previous studies have suggested that it is not the quantity of IFN-γ or IL-4 production that is important in determining protective immunity to *L. major* but rather the overall T_H_1/T_H_2 balance [35], [36]. Consequently we calculated the relative T_H_1/T_H_2 balance between *L. major* infected MKP-2^−/−^ and MKP-2^+/+^ mice. In the first instance we calculated the ratio of IgG2b to IgG1 for each individual mouse, as this is a strong indicator of T_H_1/T_H_2 balance [37]–[39]. However no difference in IgG2b/IgG1 levels between *L. major* infected MKP-2^−/−^ and MKP-2^+/+^ mice were observed throughout infection (Figure 5A). Not surprisingly, the ratio increased after onset of healing toward an IgG2b bias and thus a T_H_1 response in both MKP-2^−/−^ and MKP-2^+/+^ mice. We further calculated the percentage reduction in the levels of type-1 and type-2 cytokine responses induced in MKP-2^−/−^ compared with wild-type mice following infection. MKP-2^−/−^ splenocytes from *L. major* infected mice produced, upon restimulation with *L. major* antigen-pulsed DCs, 32.6% less IFN-γ (Figure 5B) than MKP-2^+/+^ splenocytes (20.79±5.53 (SD) and 30.87±5.39 (SD) ng/ml, respectively). Under the same experimental conditions a 37.8% reduction in IL-4 production (Figure 5B) was generated by MKP-2^−/−^ splenocytes compared with MKP-2^+/+^ splenocytes (35.46±9.32 (SD) pg/ml compared with 56.99±12.13 (SD) pg/ml, respectively). Moreover, when comparing the percentage of IFN-γ/TNF-α double producing CD4^+^ T cells, as measured by flow cytometry, we found a 28.7% reduction (Figure 5B) in infected MKP-2^−/−^ compared with MKP-2^+/+^ mice. Thus MKP-2 deficiency results in a T cell hypo-responsiveness following *L. major* infection, which effects both the protective T_H_1 and the disease-exacerbating T_H_2 response to a similar degree (Figure 5C). Consequently, MKP-2^−/−^ mice have a healing phenotype similar to their wild-type counterparts.

## Discussion

In a previous study we identified a major role for MKP-2 in protecting C57BL/6 mice from infection with *L. mexicana* as MKP-2 deficiency resulted in uncontrolled lesion growth with massively increased parasite burdens [21]. Increased susceptibility was associated with MKP-2^−/−^ macrophages being inherently more susceptible than wild-type macrophages to parasite infection as a result of increased Arginase-1 expression and also reduced NO production [21]. In addition, while no specific direct defect in T cell function could be attributed to MKP-2 deficiency, a diminished parasite-specific T_H_1 and an enhanced T_H_2 response developed in MKP-2^−/−^ mice infected with *L. mexicana* [21]. Surprisingly therefore, in the present study, no differences whatsoever in the normal healing phenotype were observed between MKP-2^−/−^ and MKP-2^+/+^ mice infected with *L. major*. This was despite firstly, naïve and innately activated MKP-2^−/−^ macrophages being more permissive to *L. major* infection than wild-type macrophages which, as with *L. mexicana* infections, was associated with increased Arginase-1 and reduced iNOS activities, and secondly, a clearly reduced parasite-specific T_H_1 response in infected MKP-2^−/−^ mice.

Infection of MKP-2^−/−^ mice and their host cells with *L. major* differed to that of infection with *L. mexicana* in two significant ways that undoubtedly had profound effects on disease outcome. Firstly, in contrast to infection with *L. mexicana*, infection of MKP-2^−/−^ mice with *L. major* resulted not only in a reduced type-I response, but also in an equally reduced T_H_2 response compared with wild-type mice. Consequently there was no discernible difference in the T_H_1/T_H_2 bias between MKP-2^−/−^ and MKP-2^+/+^ mice infected with this parasite. It is well established in C57BL/6 mice, that once parasite peptide reactive-CD4^+^ and CD8^+^ T cell populations reach the proper balance in draining lymph nodes and the sites of infection, there is rapid healing, and immunity is maintained by a persistent small amastigote population in equilibrium with both effector and regulatory T cell populations [10], [40]. Furthermore studies in mice have demonstrated that the absolute amount of IFN-γ generated following infection with *L. major* did not correlate with protection or cure [35], and rather, it was the balance in the T_H_1/T_H_2 cytokine profile that was important in determining disease outcome. Secondly, classically activated MKP-2^−/−^ macrophages were equally as effective as MKP-2^+/+^ macrophages in controlling the growth of *L. major* although not, as previously demonstrated, *L. mexicana* [21]. Therefore, as the T_H_1/T_H_2 balance remained unaltered, albeit equally diminished, in *L. major* infected MKP-2^−/−^ compared with MKP-2^+/+^ mice, the comparative polarisation of classical macrophage activation would remain the same. Consequently healing takes place in MKP-2^−/−^ mice infected with *L. major* in a similar manner to infected resistant wild-type mice.

Among the intriguing questions regarding the differential outcome of *L. major* and *L. mexicana* infections in MKP-2 deficient mice is why T_H_2 responses are maintained following *L. mexicana* infection [21] but down-regulated following *L. major* infection compared with their infected wild-type counterparts? It has been demonstrated previously that during *L. major* infections high local Arginase-1 levels at the site of infection mediate L-arginine depletion, which results in impaired local CD4^+^ (and CD8^+^) T cell function, particularly IFN-γ production but also to a lesser extent IL-4 and IL-10 [28], [41]. Investigations carried out by Kropf et al. [34] using Arginase-1 expressing placenta cells and Jurkat T cells showed that Arginase-1-mediated T cell hypo-responsiveness is a consequence of the down-regulation of the CD3ζ chain, a crucial signal transducing component of the TCR [34] and significantly CD3ζ has recently been found to be down-regulated on CD4^+^ and CD8^+^ T cells along with increased Arginase-1 activity in the lesions of patients infected with *L. aethiopica* [42]. Depletion of L-arginine, as a result of the generalised elevated Arginase-1 levels in MKP-2^−/−^ mice would explain the general T cell hypo-responsiveness observed in these mice following infection with *L. major*. However, why should the T_H_2 response be maintained if not enhanced following infection of MKP-2^−/−^ mice with *L. mexicana* [21]? Studies to date clearly implicate the multiple CPB isoenzymes as important species-specific virulence factors for the *L. mexicana* complex and provide one possible explanation as to why these parasites, unlike *L. major* (reviewed by [43]) tend to induce chronic lesions in the majority of mouse strains such as the C57BL/6 strain used in this study. Not only are *L. mexicana* CPBs potent inhibitors of T_H_1 responses [44] as a consequence of disrupting macrophage signalling pathways [45], but they have also been shown to be potent inducers of IL-4 production and T_H_2 responses [46]. Consequently C57BL/6 mice infected with *L. mexicana* CPB null mutants, unlike infection with wild-type parasites, develop a healing response with reduced IL-4 production and T_H_2 responses along with elevated T_H_1 responses [47]. Thus, as a result of their highly expressed T_H_2 promoting and T_H_1 inhibiting CPBs infection of MKP-2^−/−^ mice with *L. mexicana*, unlike infection with *L. major*, is able to rescue and promote the T_H_2 component of the parasite specific T cell response which is less subject to Arginase-1 induced hypo-responsiveness than T_H_1 responses [27].

Alternative activation of both MKP-2^−/−^ and MKP-2^+/+^ macrophages resulted in increased susceptibility to *L. major*. This would support the view that the T_H_1/T_H_2 balance is important and that IL-4 and IFN-γ activities regulate each other not just at the T cell level but also at the level of the macrophage by modulation of iNOS and Arginase-1 expression. In agreement IL-4Rα signalling via macrophages/neutrophils has been shown to promote early lesion growth in *L. major* infected BALB/c mice and macrophage/neutrophil specific (LysM^cre^IL-4Rα^−/lox^) IL-4Rα^−/−^ mice display delayed lesion growth [16]. The control of *L. major* early in infection in LysM^cre^IL-4Rα^−/lox^ mice has been identified as being due to enhanced macrophage microbicidal NO mediated activity in the absence of alternative macrophage activation. Paradoxically we failed to identify any disease promoting contributory role for IL-4Rα signalling via macrophages/neutrophils during *L. mexicana* infection [48]. In agreement in the present studies we also failed to identify a disease promoting role for IL-4 in either MKP-2^−/−^ or MKP-2^+/+^ macrophages infected with *L. mexicana* (Figure S2). Thus the interaction of *L. major* and *L. mexicana* with their host macrophages must be significantly different. What may be critical in this regard is that *L. amazonensis* parasites, which belong to the “*mexicana”* complex of parasites, have been shown to be more resistant to macrophage-mediated control than *L. major* requiring higher levels of NO to induce killing [49], [50]. The evidence would suggest that macrophage killing of “*mexicana*” complex parasites unlike *L. major* requires NO and additionally, superoxide [51]. Furthermore, recent evidence indicates that, unlike *L. major*, there is in fact enhanced replication of the amastigote stage of *L. amazonensis* in IFN-γ-stimulated murine macrophages despite higher NO production [52], reportedly due to the induction of a novel L-arginine pathway independent of iNOS or host Arginase-1 [53]. Induction of Arginase-1 by *L. amazonensis* has also been shown to enhance replication of the amastigote stage of the parasite [52], [53] while inhibition studies have shown that the enhanced susceptibility of MKP-2^−/−^ macrophages for *L. mexicana* is associated with enhanced Arginase-1 expression [21]. Thus given the relatively higher sensitivity of *L. major* to NO matched with the increased importance of Arginase-1 to infection with *L. mexicana* it is perhaps not surprising that classical macrophage activation ablates MKP-2 deficiency mediated differences in infectivity with the former but not the latter parasite.

Overall the present study confirms our previous observation that MKP-2 is a major factor in determining the immune response against intracellular parasites and potentially the outcome of infection. Naïve MKP-2 deficient macrophages are inherently more susceptible to *L. major* than wild-type macrophages and following infection there is a generalised T cell hypo-responsiveness. However, despite these apparent deficiencies the disease phenotype of MKP-2^−/−^ mice following *L. major* infection does not differ from wild-type mice. Our results suggest that this is a consequence of the T_H_1/T_H_2 balance remaining unaltered in MKP-2^−/−^ mice infected with *L. major* and that classical macrophage activation suffices to ablate the innate permissiveness of MKP-2^−/−^ macrophages to this species.

## Supporting Information

Figure S1 **Arginase-1 levels are elevated in spleen and intraperitoneal exudates of MKP-2 deficient mice.** Equal concentrations of whole cell lysates of intraperitoneal (IP)-washes (left panel) and spleen (right panel) of three mice were run on 12% gels, blotted on nitrocellulose membranes and stained for Arginase-1. No Arginase-1 was detected in lymph nodes (not shown). Samples were also run on 7.5% SDS gels, blotted and stained for iNOS. However, iNOS could not be detected in any of the samples (not shown).
(TIF)

Figure S2 **External addition of IL-4 does not drastically increase intracellular growth of** ***L. mexicana*** **.** Bone marrow-derived macrophages of MKP-2^−/−^ (open columns) and MKP-2^+/+^ (closed columns) mice grown on cover slips have been infected with *L. mexicana* promastigotes at a multiplicity of infection (MOI) of 5. One hour after infection free parasites have been washed off and medium was replaced with complete RPMI supplemented with or without 100 U/ml IL-4 and incubated for 48 h at 34°C. Macrophages were fixed in methanol and stained with Giemsa. Intracellular parasites were counted in a total of 200 macrophages using a bright field microscope and are shown as number of parasites per 100 macrophages. The mean percentage of infected macrophages is also expressed as numbers inside the graphs. Error bars show standard error of the mean (SEM).
(TIF)
