# Supplementary figures and images for: T Cell Hypo-Responsiveness against Leishmania major in MAP Kinase Phosphatase (MKP) 2 Deficient C57BL/6 Mice Does Not Alter the Healer Disease Phenotype
Source: PLoS Negl Trop Dis. 2013 Feb 21;7(2):e2064. doi: 10.1371/journal.pntd.0002064 (PMC3578781; doi:10.1371/journal.pntd.0002064)

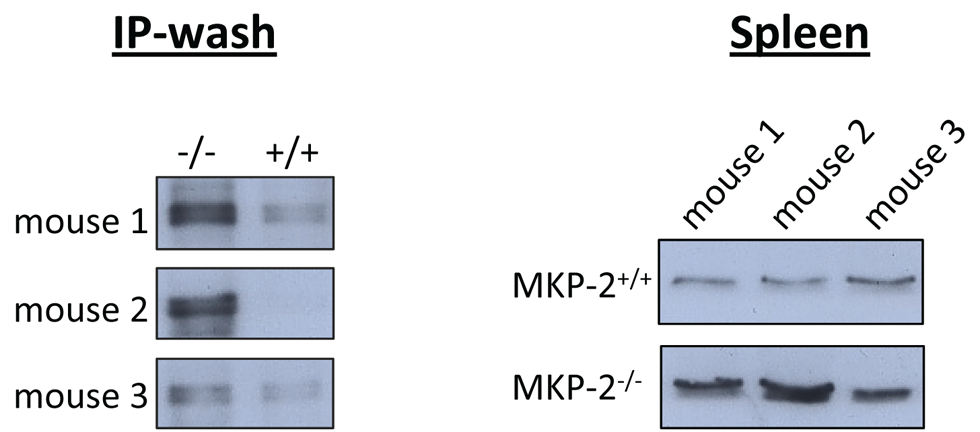

Supplement: Figure S1 — Arginase-1 levels are elevated in spleen and intraperitoneal exudates of MKP-2 deficient mice. Equal concentrations of whole cell lysates of intraperitoneal (IP)-washes (left panel) and spleen (right panel) of three mice were run on 12% gels, blotted on nitrocellulose membranes and stained for Arginase-1. No Arginase-1 was detected in lymph nodes (not shown). Samples were also run on 7.5% SDS gels, blotted and stained for iNOS. However, iNOS could not be detected in any of the samples (not shown). (TIF) [file pntd.0002064.s001.tif]

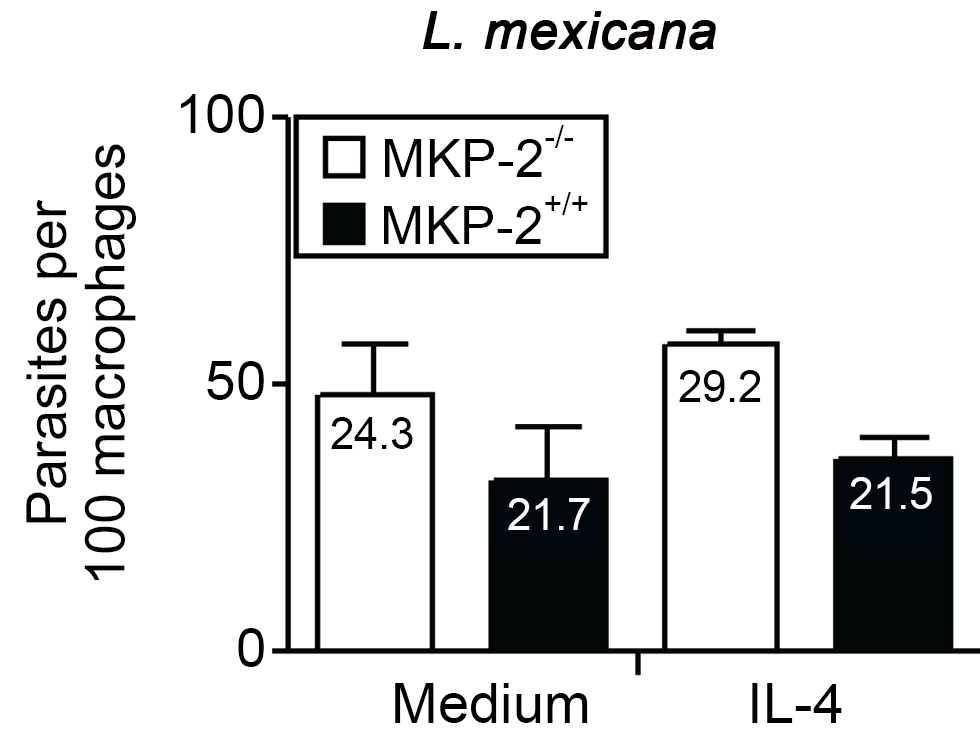

Supplement: Figure S2 — External addition of IL-4 does not drastically increase intracellular growth of L. mexicana . Bone marrow-derived macrophages of MKP-2−/− (open columns) and MKP-2+/+ (closed columns) mice grown on cover slips have been infected with L. mexicana promastigotes at a multiplicity of infection (MOI) of 5. One hour after infection free parasites have been washed off and medium was replaced with complete RPMI supplemented with or without 100 U/ml IL-4 and incubated for 48 h at 34°C. Macrophages were fixed in methanol and stained with Giemsa. Intracellular parasites were counted in a total of 200 macrophages using a bright field microscope and are shown as number of parasites per 100 macrophages. The mean percentage of infected macrophages is also expressed as numbers inside the graphs. Error bars show standard error of the mean (SEM). (TIF) [file pntd.0002064.s002.tif]
